# Phthalate Levels in Cord Blood Are Associated with Preterm Delivery and Fetal Growth Parameters in Chinese Women

**DOI:** 10.1371/journal.pone.0087430

**Published:** 2014-02-04

**Authors:** Yujing Huang, Junnan Li, Jose M. Garcia, Hui Lin, Yanzhou Wang, Ping Yan, Lingqiao Wang, Yao Tan, Jiaohua Luo, Zhiqun Qiu, Ji-an Chen, Weiqun Shu

**Affiliations:** 1 Department of Environmental Hygiene, College of Preventive Medicine, Third Military Medical University, Chongqing, China; 2 Department of gynecology and obstetrics, Southwest Hospital, Third Military Medical University, Chongqing, China; 3 Division of Endocrinology, Center for Translational Research in Inflammatory Diseases, Michael E. DeBakey VAMC/Baylor College of Medicine, Houston, Texas, United States of America; 4 Department of Epidemiology, College of Preventive Medicine, Third Military Medical University, Chongqing, China; University of Cincinnati, United States of America

## Abstract

Data concerning the effects of phthalate exposure on preterm delivery and fetal growth are limited in humans. In this paper, we assessed the relationship between 15 phthalate levels in cord blood and preterm delivery and fetal growth parameters in 207 Chinese women going into labor. Exposure to phthalates except DCHP was associated with gestational age reduction and preterm delivery (p<0.05). There were associations between phthalates and fetal growth parameters, many of which disappeared when analyses were adjusted for gestational age, especially in male infants (Only DEEP was associated with birth weight; DEP, DNHP, BBP, DNP with abdominal circumference; DEP, DBP, DCHP, DEHP with femur length in female infants. And DPP, DBEP was associated with birth length in male infants. p<0.05). This study indicates that prenatal exposure to phthalates is associated with younger gestational age and preterm delivery. Also, phthalate exposure may adversely affect fetal growth parameters via gestational age reduction and preterm delivery with a significant gender effect.

## Introduction

Phthalate Acid Esters (PAEs) are a class of synthetic chemicals which are produced in large volumes and used in a wide variety of industrial and common household products. High molecular weight phthalates (HWMP) act as additives that impart flexibility to vinyl resins. Low molecular weight phthalates (LWMP) have different uses, including in personal care products, cosmetics, food conveyor belts, artificial leather, automotive trim, traffic cones, latex adhesives, cellulose plastics and solvent for dyes [1], [2]. Because of a widespread use of phthalates in the world, phthalate compounds are commonly found in the environment [3], [4]. Some phthalates can cross the placental barrier and be detected in cord blood. Nevertheless, the effects of prenatal exposure to phthalates on fetal growth are still unknown. Some studies have suggested an association between phthalate exposure and shorter gestational age and lower birth weight [5], [6], [7], [8], [9]; but other studies found no significant relationship between prenatal exposure to phthalates and fetal growth parameters [10], [11], [12].

In our previous studies [13], [14] we analyzed organic pollutants in water samples from the Yangtze and Jialing rivers, and also phthalate levels in venous blood, cord blood, breast milk and urine from 40 healthy women undergoing parturition in Chongqing, Southwest China. More than 178 different organic pollutants were detected in the water samples. Phthalates were found in every water sample at an average concentration of 25 mg/L. Nine phthalates were detected in pregnant women. Dibutyl phthalate (DBP) was one of the chemicals frequently identified and prenatal exposure to it as measured by cord blood levels, was associated with reduced birth weight. Interestingly, levels measured in venous blood, breast milk and urine were not significantly associated with birth weight, suggesting that cord blood could be a better predictor of the risk of exposure to phthalates.

In this study we investigated the association between phthalate levels in cord blood and fetal growth, pregnancy complications, preterm delivery and other fetal growth parameters.

## Materials and Methods

### Ethical Considerations

This study was approved by the Ethics Committee of the Third Military Medical University. All participants, the children participant were presented by their parents and signed at the same consent, provide their written informed consent to participate in this study. Some consents were signed by the husbands on behalf of their wife. This consent procedure was approved by the ethics committees.

### Study Population

Study subjects included Chinese women residing in Chongqing (Southwest China) for at least two years, who delivered at Southwest Hospital in Chongqing between October 2011 and September 2012. Volunteers had no history of alcohol or tobacco use, were 18–35 years old, had undergone prenatal examination at the department of gynecology and obstetrics, Southwest Hospital, and had no family or personal history of occupational exposure to phthalates. Gestational age was determined by the last menstrual period. We recruited 207 consecutive volunteers meeting the inclusion/exclusion criteria mentioned above, 33 of which had preterm delivery (defined as a delivery with gestational age less than 37 weeks). A questionnaire was administered to participants after labor to obtain information on socio-demographic characteristics, medical history and lifestyle factors. Delivery characteristics and fetal growth parameters were obtained from the perinatal database of Southwest Hospital in Chongqing where the patients delivered. This included presence of premature rupture of membranes (PROM), chorioamnionitis, intrahepatic cholestasis of pregnancy (ICP), placenta previa, pregnancy-induced hypertension syndrome (PIH, including preeclampsia), gestational diabetes mellitus (GDM), abruption placentae, intravenous infusions therapy history (within the last week before labor), gestational age, birth weight, birth length, head circumference (HC), biparietal diameter (BPD), abdominal circumference (AC) and femur length (FL). Five ml of cord blood were obtained from each subject within 10 min of delivery and stored in a heparinized glass container at −80°C until it was analyzed.

### Phthalates Measurements

A certified standard mixture of 15 phthalates (M-8061-R1; Accustandard Inc., New Haven, CT, USA) including Dimethyl phthalate (DMP), Diethyl phthalate (DEP), Diisobutyl phthalate (DIBP), Dibutyl phthalate (DBP), bis (2-methoxyethyl) phthalate (DMEP), bis (4-methyl-2-pentyl) phthalate (BMPP), bis (2-ethoxyethyl) phthalate (DEEP), Diamyl phthalate (DPP), Dihexyl phthalate (DNHP), Benzyl butyl phthalate (BBP), bis (2-n- butoxyethyl) phthalate (DBEP), Dicyclohexyl phthalate (DCHP), bis (2-ethyl hexyl) phthalate (DEHP), Di-n-octyl phthalate (DNOP) and Dinonyl phthalate (DNP) was used as a calibration standard.

Phthalates in cord blood were extracted according to the method described by Hogberg et al [15]. Briefly, the samples (5 ml cord blood were used to extract phthalates as this amount was enough for detection according to our previous research [13]) were thawed and diluted with ultrapure water (1∶1). The sample was extracted twice with 5 mL hexane: MTBE (1∶1) for 30 min and once with 3 mL hexane for 15 min. The extract was analyzed by Gas Chromatography/Mass Spectrometry (GC/MS; Agilent 7890A\5975C; Agilent Technologies Inc., Santa Clara, CA, USA). Phthalates were identified and quantitated by their characteristic retention time, quantification ion and confirmation ion. At least two blanks (ultrapure water stored in a heparinized glass container at −80°C) and two positive controls (ultrapure water containing the calibration standard stored in a heparinized glass container at −80°C) were analyzed along with each batch of samples to monitor background concentrations of phthalates.

### Statistical Analyses

All statistical analyses were performed with SPSS for windows version 18.0. Independent sample T-tests and Chi-square tests were used to analyze the characteristics of pregnant women in the preterm and term delivery groups and their offspring. Linear regression and independent sample T-test were used to estimate the association between phthalates and gestational age and fetal growth parameters, and binary logistic regression and Chi-square test were used to estimate the association between phthalates and preterm delivery. Normal distribution was confirmed in all tests performed by one sample Kolmogorov-Smirnov Test.

For linear regression and binary logistic regression analyses, we replaced the concentration below the limits of detection (LOD) by LOD/√2 when the detection rate of the phthalate was more than 60% and transformed them into Ln-transformation for analyses. When the detection rate was less than 60%, the concentrations were converted into categorical variables by replacing the values for 1 (detected) or 0 (undetected).

For independent sample T-test and Chi-square test analyses, pregnant women were graded from low cord blood PAEs concentration to high cord blood PAEs concentration by the phthalates level (dividing at the median when the detection rate was more than 50% and at the LOD when the detection rate was less than 50%).

## Results

### Characteristics of Pregnant Women and Their Offspring

Table 1 presents the characteristics of pregnant women and their offspring in this study. The mean age for the 207 women included in the study was 28 years old. Thirty-three of them had a preterm delivery. There were no significant differences between the term and preterm groups for maternal age, BMI before pregnancy, prenatal examination, pregnancy history and rate of most pregnancy related diseases except PIH and PROM which were higher in the preterm group. Birth length and weight, BPD, HC, AC, FL were significantly lower in the preterm group (p<0.05). All 15 phthalates were detected in cord blood samples, and levels of DBP, DIBP, DEHP were detected in every sample. The LOD ranged from 0.04 µg/l to 0.31 µg/l and the recovery rate ranged from 74.70% to 93.85% (Table 2).

**Table 1:** Characteristics of pregnant women and their offspring.

Characteristic	Mean± SD or N(%)			*p*
	Total (N = 207)	Preterm Group (N = 33)	Term Group (N = 174)	
Maternal age(years)	28.06±3.28	27.45±3.61	28.17±3.22	0.25^a^
BMI (kg/m^2^)	20.82±2.82	21.41±3.61	20.71±2.64	0.30^a^
Prenatal examination	207 (100.00)	33(100)	174(100)	1.000^b^
Pregnancy history				0.450^b^
First child	102 (49.28)	14(42.42)	88(50.57)	
Not first child	105 (50.72)	19(57.58)	86(49.43)	
PROM	59(28.50)	19(57.58)	40(22.99)	<0.001^b^
Chorioamnionitis	2(0.97)	0(0)	2(1.15)	1.000^b^
ICP	4(1.93)	2(6.06)	2(1.15)	0.120^b^
Placenta previa	3(1.45)	1(3.03)	2(1.15)	0.408^b^
PIH	7(3.38)	5(15.15)	2(1.15)	0.001^b^
GDM	45(21.74)	9(27.27)	36(20.69)	0.489^b^
Abruption placentae	1(0.48)	1(3.03)	0(0)	0.159^b^
IV therapy.	53(25.60)	18(54.50)	35(20.10)	<0.001^b^
Placental weight (g)	535.09±81.35	502.06±118.39	539.25±75.02	0.076^a^
Placental volume (cm^3^)	456.33±168.45	520.98±315.22	444.26±121.28	0.191^a^
Gestational age (weeks)	38.57±2.05	34.85±1.60	39.28±1.18	<0.001^a^
Infant sex				1.000^b^
Male	95 (45.89)	15(45.45)	80(45.98)	
Female	112 (54.11)	18(54.55)	94(54.02)	
Birth length (cm)	49.50±2.58	46.15±3.78	50.14±1.66	<0.001^a^
Birth weight (g)	3207.14±488.05	2481.02±519.37	3344.86±337.77	<0.001^a^
AC (mm)	329.33±20.06	301.55±29.97	333.81±13.54	<0.001^a^
FL (mm)	70.04±4.03	64.52±5.13	70.91±3.04	<0.001^a^
HC (mm)	326.78±14.21	312.17±19.22	329.44±11.40	0.010^a^
BPD (mm)	92.77±4.17	88.18±6.23	93.59±3.05	<0.001^a^

a estimated using independent samples T – test.

b estimate using chi - square test. PROM: premature rupture of membranes. ICP: intrahepatic cholestasis of pregnancy. PIH: pregnancy induced hypertension syndrome (including preeclampsia). GDM: gestational diabetes mellitus. HC: head circumference. BPD: biparietal diameter. AC: abdominal circumference. FL: femur length. BMI: body mass index. IV: Intravenous.

**Table 2:** Cord blood concentrations of 15 PAEs.

Analyte	Recovery rate (%)	LOD (µg/L)	Detection (%)	mean (µg/L)	5 th	25 th	50 th	75 th	95 th
DMP (µg/L)	74.70	0.06	51(24.64)	6.69	N.D.	N.D.	N.D.	N.D.	11.30
DEP (µg/L)	85.35	0.09	68(32.85)	8.99	N.D.	N.D.	N.D.	0.73	18.95
DMEP (µg/L)	90.01	0.11	126(60.87)	8.11	N.D.	N.D.	3.72	7.08	19.25
DBP (µg/L)	76.74	0.06	207(100.00)	68.14	10.10	19.61	36.21	72.03	265.40
DEEP (µg/L)	81.66	0.04	119(57.49)	32.96	N.D.	N.D.	3.84	9.17	103.32
DIBP (µg/L)	87.32	0.04	207(100.00)	31.34	6.49	11.08	16.69	26.92	114.14
DPP (µg/L)	93.85	0.05	38(18.36)	26.64	N.D.	N.D.	N.D.	N.D.	31.79
BMPP (µg/L)	92.13	0.25	41(19.81)	11.81	N.D.	N.D.	N.D.	N.D.	17.43
DBEP (µg/L)	88.84	0.31	150(72.46)	53.51	N.D.	N.D.	1.36	4.64	109.57
DCHP (µg/L)	91.86	0.05	184(88.89)	125.02	N.D.	5.83	13.67	38.46	384.38
DNHP (µg/L)	80.56	0.05	59(28.50)	8.08	N.D.	N.D.	N.D.	0.29	9.66
BBP (µg/L)	87.75	0.15	95(45.89)	22.55	N.D.	N.D.	N.D.	0.99	89.87
DEHP (µg/L)	79.32	0.06	207(100.00)	187.16	3.12	9.18	19.70	78.46	841.16
DNOP (µg/L)	92.07	0.08	61(29.47)	27.66	N.D.	N.D.	N.D.	0.44	61.26
DNP (µg/L)	92.59	0.13	173(83.57)	13.42	N.D.	0.23	0.68	2.13	71.85

N.D. (not detected) indicates analyte below the level of detection.

### Cord Blood Phthalate Levels and Preterm Delivery

Cord blood levels of all 15 phthalates predicted gestational age reduction (*p*<0.05, Table 3) and preterm delivery (except DCHP, *p*<0.05, Table 3, Figure S1). A history of intravenous infusions therapy was associated with preterm delivery (*p*<0.01, Table 1) and 9 phthalates (DMP, DEP, DPP, BMPP, DNHP, BBP, DNOP, DBP and DEHP, *p*<0.05, Figure S2). But it did not affect the relationship between cord blood levels of phthalates and preterm delivery (Table 3).

**Table 3:** Association between cord blood PAEs levels and gestational age and preterm delivery.

Analyte	Gestational Age (weeks)		Preterm Delivery			
	β_i_ c (95% C.I.) (unadjusted,n = 207)	β_a_ d (95% C.I.) (adjusted,n = 207)	OR_i_ e (95% C.I.) (unadjusted,n = 207)	OR_a_ f (95% C.I.) (adjusted, n = 207)	OR_u_ g (95% C.I.) (adjusted in no IV group, n = 154)	OR_u_ h (95% C.I.) (adjusted in IV group, n = 53)
DMP^a^	−2.48**(−3.04, −1.92)	−2.22**(−2.80, −1.65)	36.77**(12.89,104.83)	34.47**(11.32,104.94)	0.01**(0.00, 0.09)	0.06**(0.02, 0.24)
DEP^a^	−1.93**(−2.47, −1.39)	−1.67**(−2.23, −1.12)	18.76**(6.80,51.77)	16.13**(5.63,46.21)	0.02**(0.00, 0.25)	0.11**(0.03, 0.38)
DEEP^a^	−1.10**(−1.65, −0.55)	−1.01**(−1.54, −0.48)	15.15**(3.52,65.26)	15.14**(3.44,66.66)	0.03**(0.00, 0.30)	0.08*(0.01, 0.63)
DPP^a^	−3.22**(−3.80, −2.64)	−3.00**(−3.61, −2.40)	66.68**(22.76,195.35)	58.34**(18.78,181.24)	0.01**(0.00, 0.10)	0.03**(0.01, 0.11)
BMPP^a^	−2.91**(−3.49, −2.32)	−2.68**(−3.27, −2.09)	39.37**(14.65,105.78)	50.05**(15.62,160.36)	0.01**(0.00, 0.14)	0.03**(0.01, 0.12)
DNHP^a^	−1.99**(−2.55, −1.43)	−1.67**(−2.26, −1.09)	19.97**(7.61,52.37)	16.22**(5.93,44.38)	0.04**(0.01, 0.28)	0.09**(0.02, 0.32)
BBP^a^	−1.32**(−1.85, −0.78)	−1.05**(−1.59, −0.51)	11.86**(3.99,35.26)	9.97**(3.25,30.53)	0.06*(0.01, 0.58)	0.16**(0.04, 0.63)
DNOP^a^	−2.14**(−2.68, −1.60)	−1.89**(−2.45, −1.34)	23.93**(8.59,66.64)	20.20**(7.01,58.18)	0.04**(0.01, 0.25)	0.07**(0.02, 0.26)
Ln(DMEP^b^)	−0.28**(−0.40, −0.16)	−0.24**(−0.36, −0.12)	2.18**(1.47,3.23)	1.99**(1.37,2.89)	2.05**(1.23, 3.44)	1.80*(1.13, 2.88)
Ln(DBP^b^)	−0.70**(−0.94, −0.46)	−0.55**(−0.81, −0.30)	3.83**(2.38,6.16)	3.35**(2.05,5.50)	2.38*(1.01, 5.61)	3.60**(1.82, 7.12)
Ln(DIBP^b^)	−0.90**(−1.17, −0.64)	−0.75**(−1.03, −0.46)	6.54**(3.63,11.77)	6.01**(3.24,11.17)	4.78**(1.68, 13.57)	6.07**(2.66, 13.83)
Ln(DBEP^b^)	−0.48**(−0.61, −0.36)	−0.42**(−0.55, −0.28)	2.63**(1.95,3.56)	2.56**(1.87,3.51)	3.12**(1.63, 5.97)	2.25**(1.56, 3.24)
Ln(DCHP^b^)	−0.15*(−0.26, −0.04)	−0.10(−0.22,0.01)	1.27*(1.05,1.53)	1.22(0.99,1.50)	0.95(0.66, 1.38)	1.30(0.98, 1.73)
Ln(DEHP^b^)	−0.54**(−0.68, −0.40)	−0.46**(−0.61, −0.31)	2.41**(1.80,3.24)	2.32**(1.71,3.16)	2.05*(1.19, 3.54)	2.26**(1.54, 3.30)
Ln(DNP^b^)	−0.55**(−0.66, −0.43)	−0.49**(−0.62,−0.37)	2.43**(1.90,3.12)	2.34**(1.80,3.04)	2.43**(1.52, 3.90)	2.13**(1.54, 2.93)

a Concentrations were converted into categorical variables as detectable and non-detectable.

b Concentrations below the level of detection (LOD) were replaced by LOD/√2 and transformed into Ln- transformation.

c Unadjusted regression coefficients for linear regression analyses.

d Regression coefficients for linear regression analyses adjusted for maternal age, BMI, frequency of prenatal examination, history of intravenous infusions therapy and pregnancy history.

e Unadjusted odds ratios from binary logistic regression analyses.

f Odds ratios from binary logistic regression analyses adjusted for maternal age, BMI, frequency of prenatal examination and pregnancy history.

g Odds ratio from binary logistic regression analyses adjusted for maternal age, BMI, frequency of prenatal examination and pregnancy history in the group with no history of intravenous infusions therapy.

h Odds ratio from binary logistic regression analyses adjusted for maternal age, BMI, frequency of prenatal examination and pregnancy history in the group with a history of intravenous infusions therapy.

* *p*<0.05.

** *p*<0.01.

### Cord Blood Phthalate Levels and Fetal Growth Parameters

In female infants, 14 phthalates except DCHP were significantly associated with decreased birth weight (*p*<0.05, Table 4); 13 phthalates (DMP, DEP, DPP, BMPP, DNHP, BBP, DNOP, DMEP, DBP, DIBP, DBEP, DEHP and DNP) with decreased birth length (*p*<0.05, Table 4); 11 phthalates (DMP, DEP, DPP, DNHP, BBP, DNOP, DBP, DIBP, DBEP, DEHP and DNP) with decreased abdominal circumference (*p*<0.05, Table 4); 11 phthalates (DMP, DEP, DPP, BMPP, DNHP, DBP, DIBP, DBEP, DCHP, DEHP and DNP) with decreased femur length (*p*<0.05, Table 5); 13 phthalates (DMP, DEP, DPP, BMPP, DNHP, BBP, DNOP, DBP, DIBP, DBEP, DCHP, DEHP and DNP) with decreased biparietal diameter (*p*<0.05, Table 5) and 9 phthalates (DMP, DEP, DPP, DNHP, DNOP, DBP, DIBP, DEHP and DNP) with decreased head circumference (*p*<0.05, Table 5). After adjusting for gestational age, only DMEP was associated with decreased birth weight significantly (*p*<0.05, Table 4); DEP, DNHP, BBP and DNP exposure was associated with decreased abdominal circumference (*p*<0.05, Table 4); DBP, DCHP, DEHP, and DEP were associated with decreased femur length (*p*<0.05, Table 5); DNHP exposure decreased head circumference (*p*<0.05, Table 5) and none predicted birth length and biparietal diameter (*p*>0.05, Table 4, Table 5).

**Table 4:** Association between cord blood PAEs levels and fetal birth weight, birth length and Abdominal Circumference in all female infants.

Analyte	Birth Weight (g)		Birth Length (cm)		Abdominal Circumference (mm)	
	β_a1_ c (95.0% C.I.)	β_a2_ d (95.0% C.I.)	β_a1_ c (95.0% C.I.)	β_a2_ d (95.0% C.I.)	β_a1_ c (95.0% C.I.)	β_a2_ d (95.0% C.I.)
DMP^a^	−421(−592, −250)**	−78(−240,85)	−1.49(−2.48, −0.51)**	0.25(−0.76,1.25)	−13.37(−21.92, −4.82)**	−3.02(−12.81,6.77)
DEP^a^	−408(−571, −246)**	−110(−260,41)	−1.64(−2.57, −0.71)**	−0.21(−1.14,0.73)	−19.08(−26.77, −11.39)**	−12.51(−21.22, −3.81)**
DEEP^a^	−266(−436, −97)**	−143(−273, −14)*	−0.84(−1.79,0.12)	−0.26(−1.07,0.55)	−6.14(−14.53,2.24)	−1.89(−9.54,5.75)
DPP^a^	−504(−688, −320)**	−94(−281,93)	−2.03(−3.10, −0.97)**	−0.02(−1.18,1.13)	−17.99(−26.96, −9.03)**	−7.76(−18.75,3.23)
BMPP^a^	−409(−598, −220)**	−48(−221,125)	−1.79(−2.85, −0.72)**	−0.11(−1.18,0.95)	−9.34(−18.80,0.11)	1.12(−8.61,10.85)
DNHP^a^	−346(−516, −176)**	−75(−222,72)	−1.21(−2.18, −0.24)*	0.11(−0.80,1.02)	−17.49(−25.38, −9.60)**	−11.35(−19.57, −3.14)**
BBP^a^	−282(−444, −120)**	−76(−208,56)	−1.14(−2.04, −0.23)*	−0.18(−0.99,0.64)	−16.00(−23.41, −8.60)**	−11.09(−18.40, −3.78)**
DNOP^a^	−326(−499, −153)**	−42(−191,107)	−1.26(−2.23, −0.28)*	0.10(−0.82,1.01)	−10.89(−19.22, −2.56)*	−3.32(−11.76,5.13)
Ln(DMEP^b^)	−64(−100, −27)**	−28(−57,1)	−0.23(−0.43, −0.02)*	−0.06(−0.24,0.12)	−1.66(−3.43,0.10)	−0.50(−2.16,1.16)
Ln(DBP^b^)	−104(−173, −34)**	−18(−74,38)	−0.58(−0.96, −0.20)**	−0.20(−0.55,0.14)	−4.37(−7.46, −1.28)**	−1.99(−5.04,1.05)
Ln(DIBP^b^)	−139(−213, −65)**	−27(−90,36)	−0.58(−1.00, −0.17)**	−0.06(−0.45,0.33)	−4.72(−7.94, −1.50)**	−1.86(−5.15,1.43)
Ln(DBEP^b^)	−61(−105, −18)**	5(−30,41)	−0.27(−0.51, −0.03)*	0.04(−0.18,0.26)	−3.03(−5.03, −1.03)**	−1.18(−3.26,0.90)
Ln(DCHP^b^)	−15(−46,16)	9(−14,33)	−0.15(−0.32,0.02)	−0.05(−0.19,0.10)	−0.81(−2.22,0.60)	−0.14(−1.41,1.13)
Ln(DEHP^b^)	−72(−115, −29)**	−2(−39,35)	−0.46(−0.69, −0.22)**	−1.07(−0.39,0.06)	−3.46(−5.40, −1.52)**	−1.87(−3.85,0.12)
Ln(DNP^b^)	−101(−135, −66)**	−33(−67,0)	−0.41(−0.61, −0.21)**	−0.08(−0.29,0.13)	−3.71(−5.42, −2.00)**	−2.06(−4.03, −0.10)*

a Concentrations were converted into categorical variables as detectable and non-detectable.

b Concentrations below the LOD were replaced by LOD/√2 and transformed into Ln- transformation.

c Unadjusted regression coefficients from linear regression analyses.

d Regression coefficients from linear regression analyses adjusted for gestational age.

** *p*<0.01.

* *p*<0.05.

**Table 5:** Association between cord blood PAEs levels and fetal Femur Length, Biparietal Diameter and Head Circumference in all female infants.

Analyte	Femur Length (mm)		Biparietal Diameter (mm)		Head Circumference (mm)	
	β_a1_ c (95.0% C.I.)	β_a2_ d (95.0% C.I.)	β_a1_ c (95.0% C.I.)	β_a2_ d (95.0% C.I.)	β_a1_ c (95.0% C.I.)	β_a2_ d (95.0% C.I.)
DMP^a^	−2.23(−3.72, −0.73)**	−0.22(−1.76,1.31)	−2.42(−4.09, −0.75)**	−0.19(−2.00,1.61)	−14.96(−24.70, −5.23)**	−9.45(−22.10,3.20)
DEP^a^	−3.09(−4.44, −1.74)**	−1.51(−2.94, −0.07)*	−2.71(−4.28, −1.14)**	−0.74(−2.46,0.98)	−14.71(−24.72, −4.70)**	−9.07(−21.23,3.10)
DEEP^a^	−0.91(−2.37,0.56)	−0.13(−1.41,1.14)	−0.97(−2.59,0.65)	−0.08(−1.55,1.38)	−1.55(−12.60,9.49)	1.80(−8.55,12.15)
DPP^a^	−2.93(−4.55, −1.31)**	−0.71(−2.45,1.02)	−3.60(−5.36, −1.84)**	−1.41(−3.42,0.60)	−16.03(−26.13, −5.94)**	−10.61(−23.58,2.35)
BMPP^a^	−1.93(−3.56, −0.31)*	0.07(−1.52,1.66)	−1.88(−3.72, −0.05)*	0.33(−1.53,2.18)	−9.61(−21.21,2.00)	−1.63(−14.46,11.21)
DNHP^a^	−2.48(−3.91, −1.05)**	−1.14(−2.51,0.23)	−2.60(−4.21, −0.99)**	−1.05(−2.67,0.57)	−15.90(−25.28, −6.51)**	−11.79(−22.05, −1.52)*
BBP^a^	−1.70(−3.08, −0.33)*	−0.52(−1.80,0.75)	−2.44(−3.94, −0.94)**	−1.26(−2.71,0.19)	−9.59(−19.39,0.22)	−4.07(−14.57,6.43)
DNOP^a^	−1.41(−2.92,0.09)	0.09(−1.31,1.49)	−2.38(−4.02, −0.75)**	−0.79(−2.43,0.84)	−12.51(−22.25, −2.77)*	−6.33(−18.08,5.43)
Ln(DMEP^b^)	−0.21(−0.52,0.11)	0(−0.28,0.27)	−0.22(−0.57,0.13)	0.01(−0.31,0.33)	−0.78(−3.12,1.55)	0.31(−1.97,2.58)
Ln(DBP^b^)	−0.96(−1.51, −0.41)**	−0.60(−1.10, −0.10)*	−0.71(−1.35, −0.08)*	−0.25(−0.85,0.36)	−6.25(−10.82, −1.68)**	−3.87(−8.97,1.23)
Ln(DIBP^b^)	−0.94(−1.54, −0.33)**	−0.39(−0.96,0.19)	−0.85(−1.55, −0.16)*	−0.21(−0.89,0.47)	−6.56(−11.18, −1.93)**	−3.85(−9.47,1.76)
Ln(DBEP^b^)	−0.53(−0.87, −0.18)**	−0.16(−0.49,0.18)	−0.47(−0.87, −0.08)*	−0.05(−0.44,0.35)	−1.89(−4.62,0.85)	0.09(−2.90,3.09)
Ln(DCHP^b^)	−0.41(−0.65, −0.18)**	−0.27(−0.48, −0.05)*	−0.32(−0.59, −0.04)*	−0.17(−0.42,0.09)	−1.59(−3.19,0.01)	−1.10(−2.64,0.44)
Ln(DEHP^b^)	−0.79(−1.12, −0.46)**	−0.48(−0.81, −0.15)**	−0.66(−1.06, −0.27)**	−0.30(−0.69,0.10)	−3.33(−5.59, −1.08)**	−2.27(−4.73,0.18)
Ln(DNP^b^)	−0.55(−0.86, −0.24)**	−0.17(−0.48,0.15)	−0.65(−0.99, −0.31)**	−0.26(−0.63,0.12)	−3.22(−5.22, −1.22)**	−2.20(−4.64,0.24)

a Concentrations were converted into categorical variables as detectable and non-detectable.

b Concentrations below the LOD were replaced by LOD/√2 and transformed into Ln- transformation.

c Unadjusted regression coefficients from linear regression analyses.

d Regression coefficients from linear regression analyses adjusted for gestational age.

** *p*<0.01.

* *p*<0.05.

In male infants, 13 phthalates (DMP, DEP, DEEP, DPP, BMPP, DNHP, DNOP, DMEP, DBP, DIBP, DBEP, DEHP and DNP) were significantly associated with decreased birth weight (*p*<0.05, Table 6); 13 phthalates (DMP, DEP, DEEP, DPP, BMPP, DNHP, DNOP, DMEP, DBP, DIBP, DBEP, DEHP and DNP) with decreased birth length (*p*<0.05, Table 6); 11 phthalates (DMP, DEP, DEEP, DPP, BMPP, DNHP, BBP, DNOP, DBP, DIBP, DBEP, DEHP and DNP) with decreased abdominal circumference (*p*<0.05, Table 6); 9 phthalates (DMP, DPP, BMPP, DNOP, DBP, DIBP, DBEP, DEHP and DNP) with decreased femur length (*p*<0.05, Table 7); 10 phthalates (DMP, DPP, BMPP, DNHP, DNOP, DBP, DIBP, DBEP, DEHP and DNP) with decreased biparietal diameter (*p*<0.05, Table 7); 5 phthalates (DPP, BMPP, DNOP, DIBP and DBEP) with decreased head circumference (*p*<0.05, Table 7). However, after adjusting for gestational age, only DPP and DBEP decreased birth length significantly (*p*<0.05, Table 6); none predicted birth weight, abdominal circumference, femur length, head circumference and biparietal diameter (*p*>0.05, Table 6, Table 7).

**Table 6:** Association between cord blood PAEs levels and fetal birth weight, birth length and Abdominal Circumference in all male infants.

Analyte	Birth Weight (g)		Birth Length (cm)		Abdominal Circumference (mm)	
	β_a1_ c (95.0% C.I.)	β_a2_ d (95.0% C.I.)	β_a1_ c (95.0% C.I.)	β_a2_ d (95.0% C.I.)	β_a1_ c (95.0% C.I.)	β_a2_ d (95.0% C.I.)
DMP^a^	−554(−799, −309)**	−96(−318,126)	−2.82(−4.08, −1.56)**	−0.85(−2.10,0.41)	−18.97(−31.58, −6.36)	−3.51(−14.83,7.81)
DEP^a^	−313(−536, −90)**	−17(−194,160)	−1.67(−2.82, −0.53)**	−0.37(−1.37,0.64)	−10.64(−22.26,0.98)	−0.41(−9.92,9.10)
DEEP^a^	−272(−481, −64)*	−8(−172,155)	−1.37(−2.45, −0.30)*	−0.19(−1.12,0.74)	−10.55(−21.05, −0.04)*	−1.89(−10.47,6.69)
DPP^a^	−830(−1082, −577)**	−249(−528,31)	−4.23(−5.53, −2.93)**	−1.92(−3.49, −0.36)*	−31.39(−45.49, −17.29)**	−8.21(−23.29,6.87)
BMPP^a^	−734(−980, −488)**	−135(−405,135)	−3.67(−4.95, −2.40)**	−1.20(−2.72,0.32)	−28.89(−42.53, −15.26)**	−3.89(−18.98,11.21)
DNHP^a^	−481(−713, −249)**	−102(−302,97)	−2.62(−3.79, −1.44)**	−1.01(−2.13,0.11)	−13.18(−25.30, −1.06)*	−0.16(−10.45,10.12)
BBP^a^	−101(−318,116)	128(−30,287)	−0.55(−1.67,0.56)	0.48(−0.42,1.39)	−4.43(−15.24,6.38)	4.37(−4.15,12.89)
DNOP^a^	−415(−642, −188)**	39(−162,241)	−2.21(−3.36, −1.05)**	−0.26(−1.41,0.88)	−19.60(−31.80, −7.40)**	0.67(−11.35,12.70)
Ln(DMEP^b^)	−52(−100, −5)*	7(−30,44)	−0.34(−0.58, −0.10)**	−0.09(−0.29,0.12)	−1.04(−3.55,1.46)**	0.96(−1.01,2.93)
Ln(DBP^b^)	−160(−265, −55)**	10(−76,97)	−0.98(−1.50, −0.45)**	−0.26(−0.75,0.23)	−7.04(−12.43, −1.66)*	−1.64(−6.22,2.95)
Ln(DIBP^b^)	−286(−409, −163)**	−87(−195,20)	−1.58(−2.20, −0.96)**	−0.75(−1.35, −0.15)	−10.51(−16.63, −4.39)**	−3.53(−9.01,1.95)
Ln(DBEP^b^)	−122(−168, −75)**	−32(−76,12)	−0.66(−0.90, −0.43)**	−0.30(−0.54, −0.05)*	−5.01(−7.40, −2.62)**	−1.70(−4.04,0.64)
Ln(DCHP^b^)	−37(−88,14)	−15(−51,22)	−0.23(−0.49,0.03)	−0.13(−0.34,0.07)	−2.25(−4.58,0.08)	−1.31(−3.11,0.50)
Ln(DEHP^b^)	−120(−181, −58)**	−1(−55,54)	−0.60(−0.92, −0.29)**	−0.08(−0.39,0.23)	−4.72(−7.79, −1.65)*	−0.87(−3.65,1.90)
Ln(DNP^b^)	−119(−170, −69)**	−16(−64,31)	−0.61(−0.87, −0.35)**	−0.17(−0.44,0.10)	−4.84(−7.45, −2.22)**	−1.12(−3.64,1.41)

a Concentrations were converted into categorical variables as detectable and non-detectable.

b Concentrations below the LOD were replaced by LOD/√2 and transformed into Ln- transformation.

c Unadjusted regression coefficients from linear regression analyses.

d Regression coefficients from linear regression analyses adjusted for gestational age.

** *p*<0.01.

* *p*<0.05.

**Table 7:** Association between cord blood PAEs levels and fetal Femur Length, Biparietal Diameter and Head Circumference in all male infants.

Analyte	Femur Length (mm)		Biparietal Diameter (mm)		Head Circumference (mm)	
	β_a1_ c (95.0% C.I.)	β_a2_ d (95.0% C.I.)	β_a1_ c (95.0% C.I.)	β_a2_ d (95.0% C.I.)	β_a1_ c (95.0% C.I.)	β_a2_ d (95.0% C.I.)
DMP^a^	−3.06(−5.65, −0.47)*	−0.23(−2.84,2.39)	−3.08(−5.40, −0.77)*	0.15(−2.15,2.46)	−0.77(−10.47,8.94)	4.95(−4.83,14.73)
DEP^a^	−1.62(−3.98,0.74)	0.31(−1.87,2.50)	−1.12(−3.23,0.99)	1.25(−0.63,3.13)	2.99(−4.80,10.77)	4.63(−2.57,11.83)
DEEP^a^	−1.03(−3.15,1.09)	0.77(−1.18,2.71)	−1.12(−3.05,0.80)	0.68(−1.01,2.38)	−4.58(−11.75,2.53)	−1.95(−9.12,5.22)
DPP^a^	−5.63(−8.52, −2.74)**	−1.87(−5.31,1.58)	−4.85(−7.41, −2.29)**	−0.25(−3.25,2.75)	−12.88(−25.18, −0.58)*	−3.70(−21.17,13.78)
BMPP^a^	−6.13(−8.82, −3.44)**	−2.79(−6.19,0.61)	−4.54(−7.03, −2.05)**	0.35(−2.64,3.34)	−12.88(−25.18, −0.58)*	−3.70(−21.17,13.78)
DNHP^a^	−1.84(−4.32,0.64)	0.63(−1.73,2.99)	−2.28(−4.50, −0.06)*	0.48(−1.62,2.57)	0.03(−9.68,9.74)	5.92(−3.79,15.63)
BBP^a^	−1.99(−4.11,0.14)	−0.78(−2.70,1.14)	−1.00(−2.95,0.95)	0.52(−1.16,2.21)	2.42(−5.21,10.05)	4.82(−2.31,11.94)
DNOP^a^	−4.69(−6.91, −2.48)**	−2.36(−4.81,0.09	−2.65(−4.81, −0.49)*	0.84(−1.37,3.04)	−11.00(−20.74, −1.26)*	−5.47(−17.69,6.76)
Ln(DMEP^b^)	−0.19(−0.67,0.29)	0.20(−0.24,0.63)	−0.19(−0.63,0.25)	0.20(−0.18,0.58)	−0.97(−2.63,0.70)	−0.34(−2.00,1.32)
Ln(DBP^b^)	−1.85(−2.87, −0.82)*	−0.94(−1.97,0.09)	−1.37(−2.33, −0.41)**	−0.21(−1.14,0.72)	−4.03(−8.22,0.16)	−2.18(−6.66,2.31)
Ln(DIBP^b^)	−2.30(−3.56, −1.05)**	−1.12(−2.40,0.17)	−1.60(−2.77, −0.43)**	−0.09(−1.24,1.06)	−4.80(−9.12, −0.48)*	−2.76(−7.62,2.11)
Ln(DBEP^b^)	−0.88(−1.38, −0.39)**	−0.37(−0.89,0.15)	−0.71(−1.16, −0.26)**	−0.07(−0.53,0.40)	−2.66(−4.51, −0.82)**	−1.89(−4.14,0.36)
Ln(DCHP^b^)	−0.25(−0.74,0.23)	−0.09(−0.51,0.34)	−0.07(−0.53,0.39)	0.13(−0.25,0.51)	0.67(−1.20,2.54)	1.21(−0.53,2.95)
Ln(DEHP^b^)	−0.87(−1.55, −0.20)**	−0.21(−0.88,0.47)	−0.89(−1.46, −0.32)**	−0.11(−0.68,0.47)	−0.98(−3.27,1.32)	1.06(−1.62,3.73)
Ln(DNP^b^)	−1.01(−1.54, −0.48)**	−0.46(−1.03,0.10)	−0.74(−1.22, −0.27)**	−0.01(−0.51,0.49)	−0.94(−2.95,1.08)	0.83(−1.53,3.20)

a Concentrations were converted into categorical variables as detectable and non-detectable.

b Concentrations below the LOD were replaced by LOD/√2 and transformed into Ln- transformation.

c Unadjusted regression coefficients from linear regression analyses.

d Regression coefficients from linear regression analyses adjusted for gestational age.

** *p*<0.01.

* *p*<0.05.

Other adjusting factors we tested including maternal age, BMI, frequency of prenatal examination and pregnancy history did not affect the associations between PAEs exposure and fetal growth parameters in female or male infants (*p*>0.05, data not show).

There was no relationship between phthalate exposure and placental volume and weight (p>0.05, data not shown).

## Discussion

In our previous studies, DBP levels in cord blood were more significantly associated with birth weight than its levels in venous blood, breast milk and urine. Based on this we hypothesized that phthalate levels in cord blood, which avoid the effect of the placental barrier, would be associated with fetal development.

### Phthalates Exposure

All 15 phthalates were detected in cord blood samples, indicating that exposure to phthalates was very prevalent among pregnant women in Chongqing (Table 2). Although the DEHP levels were similar to levels reported previously by Zhang et al. in Shanghai, China, levels of DBP were 10-fold lower and DEP levels were 100-fold lower [9]. Both cities are located by the Yangtze River but Shanghai is located downstream of Chongqing. Also, given that Shanghai is more developed, it is expected that the rate of consumption of cosmetics (i.e. perfumes and fragrances) that use DEP as a fixative or carrier would be higher [3]. This also suggests that phthalate exposure in pregnant women may be affected by geographical and economic factors.

In our previous study we reported that DBP was generally present in pregnant women in Chongqing [13]. Here we found that 4 other phthalates in addition to DBP are also present in pregnant women with a detection rate >80%. They were DNP (83.57%), DCHP (88.89%), DIBP (100%) and DEHP (100%). These two studies were conducted in the same geographical area within a decade of each other (previous, 2002; present, 2012) and the exposure level of DBP in cord blood in the two studies was similar (52.23±32.50 µg/L in our previous study and 68.14±84.04 µg/L in the present study). This suggests an increase in the variety of phthalate pollution over the past 10 years.

### Phthalates, Gestational Age and Preterm Delivery

In our study population, all phthalates except DCHP were associated with decreased gestational age and preterm delivery.

Certain medical equipment is known to contain phthalates, and phthalate exposure is associated with intensive hospital care that occurs during preterm parturition. In china, nearly all of the preterm women underwent intravenous infusion therapy to prolong pregnancy [16], [17]. Hence, we chose a history of intravenous infusion therapy, which is associated with phthalates exposure [18], [19], [20], as an indicator of hospital care. In our study, intravenous therapy was more common during preterm parturition, and both IV therapy and preterm birth were associated with higher cord blood phthalates. However, the 15 phthalates were also elevated in the cord blood of preterm infants without a history of IV therapy. This suggests that IV therapy in the preterm group does not explain the associations between preterm birth and phthalate levels. Nevertheless it is a limitation of this study that our assessment of intensive hospital care was limited to IV therapy exposure and did not include data on other aspects of hospital care for preterm labor that could potentially add phthalates. Further studies will be needed in order to assess the effects of these factors. Phthalates and their metabolites have a half-life measured in hours and although it is possible that the time in parturition may be shorter in preterm labor than term labor and the higher preterm levels may be a function of shorter time removed from the home environment, this is unlikely because pregnant woman at our institution are not hospitalized until they are having uterine contractions or rupture of membranes.

Prenatal phthalate exposure may reduce gestational age and cause preterm delivery through other mechanisms such as activation of peroxisome proliferator activated receptor (PPARs). PPARs, a ligand-activated transcription factor that is associated with preterm labor [21], can be activated by phthalates [22], [23]. The activation of PPARs may increase the secretion of prostaglandins and matrix metalloproteinases [24], [25], which can promote uterine contraction and rupture of membranes. In our previous study DEHP inhibited the secretion of estrone, which is important in maintaining the pregnancy, by up-regulating the expression of cytochrome P450 aromatase and down-regulating the expression of 17beta-hydroxysteroid dehydrogenase through PPARγ in the ovary [26].

### Phthalates and Fetal Growth Parameters

In our study population, higher phthalate concentrations in cord blood were associated with the reduction of birth weight, birth length, AC, FL, BPD and HC, both in male and female infants. The majority of these associations lost significance after adjusting for gestational age. Moreover, gestational age were also associated with fetal growth parameters. This suggests that the adverse effects on fetal growth parameters caused by phthalates may be partially dependent on the gestational age reduction and are not a direct effect of phthalates. Also, after adjusting for gestational age, the effects phthalates were different between genders. While DNP, DCHP, DBP, DEHP, DEEP, DNHP, DEP and BBP appear to have an effect on female infants, only DNEP and DPP had an effect on male infants. These differences were also present when the term group was analyzed alone with DNP, DCHP, DBP, DEEP, DNHP, DEP and BBP affecting female infants, and DIBP and DPP affecting male infants. This data suggests that gender differences may exist in the associations between phthalates exposure and fetal growth parameters.

### Conclusions

In our study, we found an association between phthalate levels in cord blood and gestational duration and preterm delivery. Also, exposure to some phthalates was associated with adverse effects on fetal growth parameters which may partially be due to a shortened gestational duration and appears to be modulated by gender.

## Supporting Information

Figure S1 **Association between cord blood PAEs levels and the incidence of preterm delivery in all pregnant women.** The low cord blood PAEs concentration group and high cord blood PAEs concentration group were divided at the median phthalate level when the detection rate was more than 50% or at the LOD when the detection rate was less than 50%. **p<0.01.
(TIF)

Figure S2 **The proportion of high cord blood PAEs levels in intravenous infusions therapy group and non- intravenous infusions therapy group.** The high cord blood PAEs levels defines which phthalate concentration in cord blood were higher than the median phthalate level when the detection rate was more than 50% or the LOD when the detection rate was less than 50%. **p<0.01. *p<0.05.
(TIF)
