# Supplementary figures and images for: Phthalate Levels in Cord Blood Are Associated with Preterm Delivery and Fetal Growth Parameters in Chinese Women
Source: PLoS One. 2014 Feb 4;9(2):e87430. doi: 10.1371/journal.pone.0087430 (PMC3913614; doi:10.1371/journal.pone.0087430)

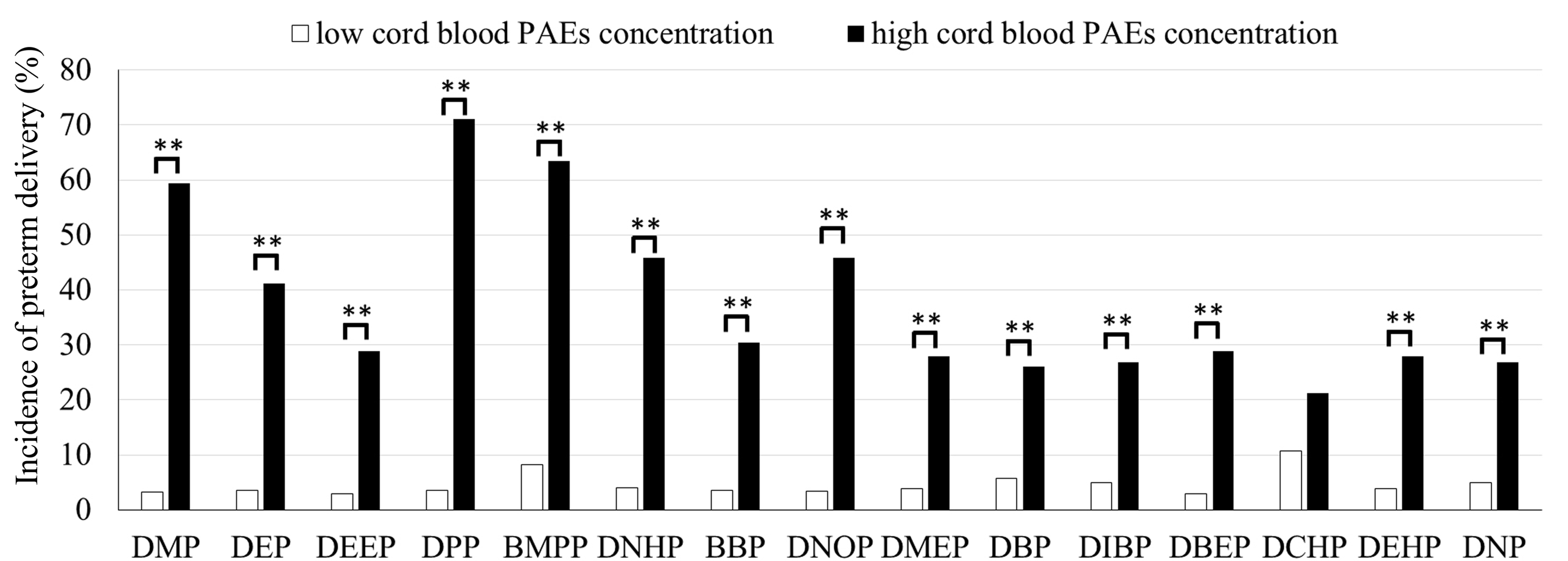

Supplement: Figure S1 — Association between cord blood PAEs levels and the incidence of preterm delivery in all pregnant women. The low cord blood PAEs concentration group and high cord blood PAEs concentration group were divided at the median phthalate level when the detection rate was more than 50% or at the LOD when the detection rate was less than 50%. **p<0.01. (TIF) [file pone.0087430.s001.tif]
